# Knowledge and Attitude of Health Professionals toward Telemedicine in Resource-Limited Settings: A Cross-Sectional Study in North West Ethiopia

**DOI:** 10.1155/2018/2389268

**Published:** 2018-11-18

**Authors:** Kirubel Biruk, Eden Abetu

**Affiliations:** ^1^Debre Markos University, College of Medicine and Health Science, Department of Health Informatics, Debre Marqos, Ethiopia; ^2^University of Gondar, College of Medicine and Health Science, Department of Clinical Pharmacy, Gondar, Ethiopia

## Abstract

**Background:**

In resource-limited environments, such as those categorized as underdeveloped countries, telemedicine becomes viewed as effective channel for utilizing the scarce medical resources and infrastructures. The aim of this study was to assess knowledge and attitude toward telemedicine among cross section of health professionals' working in three hospitals in North West Ethiopia.

**Methods:**

An institution-based cross-sectional study was conducted among 312 health professionals working in three different hospitals of North Gondar Administrative Zone during November 13 to December 10, 2017. Data were collected using structured self-administered questionnaires. Data entry and analysis were done using SPSS version 20. The mean, percentage, and standard deviation were calculated to describe the characteristics of respondents. The chi-square test was used as appropriate, to evaluate the statistical significance of the differences between the responses of the participants. A *P* value of < 0.05 was considered significant.

**Result:**

A total of 312 study subjects were approached and included in the study from November 13 to December 10, and the response rate was 95.5%. The majority of respondents (195 (65.4%)) were male, and the majority of the respondents (66.1%) were in the age group of 21–29 years. A large number of respondents (224 (75%)) were bachelor's degree holders. Only 37.6% of the respondents had demonstrated good knowledge of telemedicine, of which 74.1% were male, 65.2% of them were in the age group of 20–29 years, and 63.4% of them had >5 years of work experience. 191 (64.0%) respondents had good attitude toward telemedicine.

**Conclusion:**

The findings of the study suggest that although the respondents' knowledge of telemedicine is limited, most of them have good attitude toward telemedicine. This study underlined the need of giving training on telemedicine in order to fill the knowledge gap.

## 1. Introduction

Innovative technological advancement has caused various changes in every sector. Medical science is not an exception, and new technologies have affected both the advancement of the medicine and the way to deliver medical services [1]. This has caused a new area of health care in which medical practitioners, hospitals, health centers, and financial and medical insurance experts cooperate together in a digital environment in order to improve fairness in distribution of medical services and the quality of these services as well as reducing the costs of services. The use of information technologies in the medical and health care fields shows great potential for improving the quality and effectiveness of work done by medical organizations [2–4]. Allowing the flow of expert medical knowledge from medical, research, and teaching institutions to distant remote locations where knowledge is needed but lacking medical experts, cost, and accessibility issues are the reasons why telemedicine has been identified as possible solution to medical problems evident in underdeveloped countries [5, 6]. Despite the great promise of telemedicine, to date, its implementation in Ethiopia and other developing countries has achieved little success with low utilization [5, 7, 8]. There are possible reasons discussed in literatures that why implementation of e-health systems continues to be challenging [9–11]. As the expansion and scale-up of the telemedicine system is still ongoing in sub-Saharan Africa, a key concern of the implementation team's efforts was that physicians' and health professionals' reactions to the telemedicine system would impact implementation success. [12] The success of any new technology depends on many factors including the knowledge and understanding of the concept, skills, acquired attitude, and working environment by the concerned professionals. This is applicable for any technology-supported medical service providing methods such as telemedicine where it is important to train the new concept and assess how far they are professionally ready to accept and provide telemedicine services. Since telemedicine is an emerging technology in the health sector of Ethiopia, to facilitate the adoption, it prominently requires information about the knowledge and attitude toward telemedicine among health professionals [13–15]. This study is, therefore, aimed at assessing knowledge and attitude of health professionals regarding telemedicine.

## 2. Methods

### 2.1. Study Design and Setting

An institution-based cross-sectional study was conducted to assess the knowledge and attitude of health professionals among hospitals of North Gondar Administrative Zone, North West Ethiopia, from November 13 to December 10. North Gondar Zone has two district hospitals and one referral hospital serving a population of more than six million. During the study period, Debark hospital, University of Gondar specialized referral hospital, and Metema Hospital has 102, 833, and 136 health professionals, respectively. All health professionals who were on internship, unwilling health professionals to take part in this study, and those who were absent in the study place at the time of study were excluded.

### 2.2. Sample Size and Procedure

The sample for this study was calculated by using a single population proportion formula, with finite population correction, [16] with 95% confidence interval (CI) and a proportion of telemedicine knowledge and attitude of 50%, since there is no previous study done in the same population with a relative precision to be 5%, and 10% nonresponse rate. Accordingly, the total sample size was 312.

(1) $$\begin{array}{c} n^{\prime }=\frac{NZ^{2}P\left(1-P\right)}{d^{2}\left(N-1\right)+Z^{2}\left(P\right)\left(1-P\right)}, \end{array}$$

where *n*′ = sample size with finite population correction, *N* = population size, *Z* = statistic for a level of confidence (*Z*=1.96), *P* = expected proportion (in proportion of one) (*P*=50%), and *d* = precision (in proportion of one) (*d*=0.05).

(2) $$\begin{array}{c} n^{\prime }=\frac{\left(1071\right)1.96^{2}\left(0.5\right)\left(0.5\right)}{0.05^{2}\left(1071-1\right)+1.96^{2}\left(0.5\right)\left(0.5\right)}=283, \end{array}$$

anticipated nonrespondent rate 10% = 29, and total sample size = 283 + 29 = 312.

Proportionate stratified simple random sampling technique was performed to select study participants from those public hospitals in North Gondar Administrative Zone. The Human Resource list of health professionals in each hospital was used as a sampling frame to identify potential study participants. We assumed that all health care providers working in the same hospital were homogenous regarding knowledge and attitude toward telemedicine. Study participants were then selected randomly using record identification numbers retrieved from the sampling frame. Lottery method was used to randomly select a set of health care providers as respondents of this study.

### 2.3. Data Acquisition Instrument and Analysis

Data were collected by using structured self-administered questionnaire designed for the study, pretested on 35 health professionals of Felege Hiwot Hospital before it was distributed to research participants. The questionnaire was prepared by reviewing previous related studies [12, 17–21] and was translated to local language (Amharic) and then back to English in order to ensure that the translated version gives the proper meaning. The questionnaire consists of three main parts. Part 1 includes sociodemographic information of the participants (six items), part 2 is related to the health professionals' knowledge of telemedicine (ten items), and part 3 investigates health professionals' attitude of the relative advantages of telemedicine (seven items), compatibility of telemedicine (four items), complexity of deploying telemedicine (five items), trial ability of telemedicine application's ease of use (four items), and observability of telemedicine (three items) (see supplementary materials section for detail).

Respondents' level of knowledge of telemedicine was assessed by questions to be answered in either “Yes” or “No.” A score of “1” will be given for “Yes” and “0” for “No.” One can score a minimum of 0 and a maximum of 10 in this section. In this study, the average score of 5 (50%) from the ten questions was used as a cutoff point to determine the level of knowledge of telemedicine. The mean knowledge score less than 5 (50%) was labeled as poor knowledge of telemedicine, and more than average score of 5 (50%) was labeled as good knowledge of telemedicine.

The perceived telemedicine attributes of relative advantage, compatibility, complexity, trial ability, and observability were rated on a 5-point Likert scale that ranged from “1 = strongly disagree” to “5 = strongly agree,” except for complexity attribute questions which were reversely scored (1 = strongly agree and 5 = strongly disagree). Scores for all statements for each perceived telemedicine attribute were be averaged to create the specific mean score. In this study, mean score of less than 2.5 (50%) is labeled as poor attitude, 2.6 (51%)–3.0 (60%) as moderate, and greater than 3.0 (60%) is labeled as good attitude.

One-day training on the objective and relevance of the study, confidentiality of data, respondents' rights, informed consent, and data collection techniques was given to three individuals who were recruited to collect the data from study participants and one supervisor who conducted supportive supervision of the data collection process by the study investigators.

After collection was done, the data were checked, cleaned, edited, and analyzed by using SPSS version 20. The means and percentages were calculated to describe the profile of the respondents. The chi-square test was used as appropriate, to evaluate the statistical significance of the differences between the responses of the participants. A *P* value of <0.05 was considered significant and presented.

### 2.4. Operational Definitions

In this study, telemedicine was defined as “the delivery of healthcare services, where distance is a critical factor, by all healthcare professionals using information and communication technologies for the exchange of valid information for diagnosis, treatment and prevention of disease and injuries, research and evaluation and for the continuing education of healthcare providers, all in the interests of advancing the health of individuals and their communities” [4]. Health professionals were defined as those employees with at least a diploma certificate in the health professions who are practicing clinical service in the study settings. Good knowledge of telemedicine was defined as a score of the second part of the questionnaire more than 50%. It involves knowing the medical applications of telemedicine, knowing telemedicine infrastructure, knowing telemedicine tools (such as telesurgery and teleconsultation), and knowing the effect of telemedicine on quality of care, cost, and time. Good health professionals' attitude of telemedicine was defined as more than 50% score of the perceived relative advantage, compatibility, complexity, trial ability, and observability of telemedicine.

### 2.5. Ethical Consideration

In conducting the study, ethical clearance was secured from Department of Health Informatics, College of Medicine and Health Sciences, Debre Markos University Ethical Review Committee. Additional permissions to access participants were obtained from the offices of Hospital Directors, and verbal informed consent from the respondents was also attained.

## 3. Result

A total of 312 study subjects were approached and included in the study from November 13 to December 10, and the response rate was 95.5%. The majority of respondents (195 (65.4%)) were male, and the majority age group was between 20 and 29 years (66.1%). A large number of respondents were bachelor's degree holders (224 (75%)). Most of the study participants were nurses (158 (53.0%)), and regarding work experience, employees having less than 5 years' work experience (221 (74.2%)) were the majorities. 127 (42.6%) respondents were employees earning monthly salary of 1500–3500 Ethiopian Birr. Responses by each hospitals and respondents' sociodemographic characteristics are presented in Tables 1 and 2, respectively.

Health professionals' knowledge about telemedicine was observed from the study that 37.6% of the respondents had good knowledge of telemedicine, of which 74.1% were men, 65.2% of them were in the age group of 20–29, and 63.4% of them had >5 years of work experience. Only 22.1% (66) of the respondents have seen a telemedicine system. Most of the information sources (57.4%) about telemedicine were from colleagues of the respondents. Among physicians, 54.5% of them have good knowledge of telemedicine. Among the health professionals who were considered having good knowledge of telemedicine, 82.1% were bachelor's degree holders (Table 3 for details).

This finding indicated that health care professionals had good attitude toward telemedicine with a “relative advantage” mean of 4.1, “compatibility” mean of 3.4, and “trialability” mean of 3.8 and had poor attitude regarding the complexity and observability of telemedicine (2.4 and 2.3, respectively) as summarized in Table 4.

Among the total of 298 health professionals, 191 (64.0%) respondents had good attitude toward telemedicine. 111 (54.0%) respondents strongly agreed that telemedicine improves the quality of clinical decision, whereas 197 (66.0%) respondents agreed and strongly agreed that telemedicine threatens information confidentiality and patient privacy. 61% of health care professionals in this study appeared to know the benefit of telemedicine in saving time, and 56.4% of them knew that it reduced unnecessary transportation cost. According to the finding of this study, most health professionals (155 (52%)) thought that telemedicine is complex with 229 (76.2%) respondents opinion that telemedicine requires lots of mental effort. Sociodemographic status and health professionals' attitude toward telemedicine are shown in Table 5.

## 4. Discussion

The use of information technology in health care system is influenced by many issues. Among others, human-related components such as users' knowledge and attitude toward technology are of high importance. A survey in Michigan State University, USA, and other similar studies show that attitude and perception is an important and key research question to explain how telemedicine is viewed and conceived by health professionals [17, 18, 22–25]. To deal with these issues, targeted strategies need to be taken into consideration to facilitate the deployment of the technology.

The purpose of this study was to measure the level of knowledge and attitude of health professionals among three hospitals in North West Ethiopia. The results suggest that most of respondents had poor knowledge of various aspects of telemedicine, and even fewer had ever heard of telemedicine (36.2%), which is by far less than the number of health professionals from the study of European regions, which is 84% [26]. From those who have heard about telemedicine, the proportion of health professionals' source of information about the technology was mostly from their colleagues with 57.4%, which is as same as the study in Iran with 51.4% information source being colleagues [19]. The participants of this study had generally positive attitude toward telemedicine, but they also had significant concerns on its complexity and observability that 65.8% of them agreed and strongly agreed that telemedicine increases staff work load and 68.2% of them thought that telemedicine creates new responsibilities for the staff. These findings indicate that much work is needed to be done to educate health care professionals about telemedicine in order to lay the groundwork for successful and sustainable adoption of the technology in the country.

The proportion of respondents in this survey who have good knowledge of telemedicine (37.6%) was similar to the proportion of respondents of a cross-sectional study in India, which is about 41% [18] and slightly lower than the proportion of medical students in Sir Lanka (43%) from a survey of final-year medical students studying at the Faculty of Medicine, Sri Jayewardenepura University (SJU), Sri Lanka [27].

A study in Mashhad, Iran, in selected teaching hospitals also showed that health professionals have low knowledge about telemedicine with total awareness of health professionals with 13 ± 5.5 out of 35, which signified as low, and from those health professionals participated in the study, most of them have positive attitude toward telemedicine with total score of 63.42 ± 9.5 out of 95 (65%), which indicates a total positive attitude [19] that is almost similar with the result of this study.

Another finding in Isfahan, Iran, that 48.4% of the participants have good awareness and 63.3% of the participants have favorable attitude regarding telemedicine somehow conforms to this study [20].

Finally, this finding revealed that the majority of health professional's knowledge was low, and their attitude toward telemedicine was found to be good, which is in line with the previous results of similar studies.

## 5. Conclusion

As telemedicine is a new concept in health care service in Ethiopia, its implementation has not been as much satisfactory and successful. However, the respondents of this study confirmed positive attitude toward telemedicine. According to these findings, there is an opportunity for telemedicine to be fully integrated in the health care system in Northern Ethiopia if appropriate training and education is provided for the health care professionals. Therefore, before the deployment of this technology, it is very important to consider improving users' knowledge of the technology and demonstrate its competencies and benefits because ample knowledge and affirmative opinions of the technology are key factors to reassure users to use the technology in the future.

## 6. Limitation of the Study

The major limitation of this study was the small sample size, which was due to limited resource issue, and it was conducted among health professionals working in North Gondar Administrative Zone hospitals which were only three. Due to this reason, results may not be attributed to the whole health professional population. It would be more useful and generalizable if this study was conducted in the whole country to determine the knowledge and attitude of a larger sample of health professionals in more facilities and regions than we were able to cover.

## Figures and Tables

**Table 1:** Responses by hospital.

Hospitals	Actual no.	Received no.	Received (%)
Debark	30	27	90
Metema	39	35	89.7
Gondar university	243	236	97.1
Total response	312	298	95.5

**Table 2:** Sociodemographic characteristics of study participants.

Sociodemographic status	Number	Percent
*Gender*
Male	195	65.4
Female	103	34.6

*Age*
20–29	197	66.1
30–39	62	20.8
>39	39	13.1

*Educational status*
Diploma	44	14.8
Degree	224	75.1
Masters	22	7.4
Others^*∗*^	8	2.7

*Type of profession*		
Physician	11	3.7
Nurse	158	53.0
Health officer	13	4.4
Medical laboratory technician	38	12.8
Pharmacist	29	9.7
Midwifery	32	10.7
Others^*∗∗*^	17	5.7

*Year of experience*		
<5	221	74.2
5–10	53	17.8
>10	24	8.0

*Salary*		
<1500	22	7.4
1500–3500	127	42.6
3500–5500	84	28.2
>5500	65	21.8

^*∗*^Certificate and PhD. ^*∗∗*^Physiotherapist, psychiatrist, anesthetists, radiologist, and optometrist.

**Table 3:** Health professionals with good knowledge of telemedicine.

Sociodemographic	Total count	Percent	*P* value
*Gender*			
Male	83	74.1	0.000 *X* ^2^ = 23.181
Female	29	25.9	

*Age*			
20–29	73	65.2	0.069 (ns) *X* ^2^ = 5.351
30–39	26	23.2	
>39	13	11.6	

*Educational status*			
Diploma	9	8.0	0.000 *X* ^2^ = 18.140
Degree	92	82.1	
Masters	8	7.1	
Others^*∗*^	3	2.7	

*Type of profession*			
Physician	6	5.4	0.000 *X* ^2^ = 29.411
Nurse	65	58.0	
Health officer	4	3.6	
Medical laboratory technician	6	5.4	
Pharmacist	12	10.7	
Midwifery	8	7.1	
Others^*∗∗*^	11	9.8	

*Year of experience*			
<5	71	63.4	0.008 *X* ^2^ = 9.576
5–10	34	30.3	
>10	7	6.3	

*Salary*			
<1500	4	3.6	0.008 *X* ^2^ = 11.731
1500–3500	42	37.5	
3500–5500	39	34.8	
>5500	27	24.1	

^*∗*^Certificate and PhD. ^*∗∗*^Physiotherapist, psychiatrist, anesthetists, radiologist, and optometrist.

**Table 4:** Attitude of health professionals toward telemedicine.

Attributes of telemedicine attitude	Strongly disagree (%)	Disagree (%)	Neutral (%)	Agree (%)	Strongly agree (%)	Mean score
*Relative advantage*						
Reduce medical errors	7.0	9.4	14.1	36.9	32.6	3.8
Facilitate diagnosis and treatment	2.0	3.7	2.7	60.1	31.5	4.2
Increase communication among health care providers	1.3	2.7	4.7	56.4	34.9	4.2
Telemedicine can reduce the number of visits to health care centers	6.0	7.4	10.4	42.3	33.9	3.9
Enables me accomplish my task more quickly	3.4	7.4	16.3	37.1	35.8	3.9
Improve clinical decisions	1.0	3.0	9.4	32.6	54.0	4.4
Provide more comprehensive health care services	0.7	1.3	7.4	37.9	52.7	4.4
Total mean score						**4.1**

*Compatibility*						
In my opinion, telemedicine is compatible with all aspects of my work	8.7	14.4	10.7	41	25.2	3.6
Telemedicine is completely compatible with my current situation	10.4	11.1	11.8	38.9	27.8	3.6
I think telemedicine fits well with the way I like to work	12.1	18.1	13.4	29.6	26.8	3.4
Using telemedicine fits well into my work style	18.5	22.5	26.2	20.8	12.0	2.9
Total mean score						**3.4**

*Complexity*						
I believe using telemedicine requires a lots of mental effort^*∗*^	10.7	9.4	3.7	46.3	29.9	2.2
Learning to operate telemedicine is hard for me^*∗*^	12.8	18.7	7.9	42.1	18.5	2.7
I think telemedicine increases staff work load^*∗*^	11.1	13.0	10.1	34.9	30.9	2.4
I think telemedicine creates new responsibilities for staff^*∗*^	9.4	12.7	9.7	39.3	28.9	2.3
In my opinion, telemedicine threatens information confidentiality and patient privacy^*∗*^	9.1	11.1	13.8	28.7	37.3	2.3
Total mean score						**2.4**

*Trial ability*						
I believe to try telemedicine applications is a great opportunity	2.0	3.0	1.7	52.0	41.3	4.3
I do not have to take very much effort to try out telemedicine	11.4	16.1	22.1	27.9	22.5	3.3
I believe, using telemedicine on a trial basis is enough to see what it could do	16.1	15.8	13.1	34.5	20.5	3.3
I would like to try out telemedicine applications before using it	6.1	7.0	5.0	36.6	45.3	4.1
Total mean score						**3.8**

*Observability*						
I have seen what other hospital staffs do with telemedicine technologies	32.9	28.2	5.7	17.1	16.1	2.6
Telemedicine technology is very visible in the hospital where I work	36.2	40.0	5.0	16.1	11.7	2.4
In the hospital, I see telemedicine technology being used for many tasks	38.0	42.6	3.7	9.7	6	2.0
Total mean score						**2.3**
*Overall attitude mean score*						**3.2**

^*∗*^Reverse scored.

**Table 5:** Health professionals with good attitude toward telemedicine.

Characteristics	Number	Percent	*P* value
*Gender*			
Male	109	57.1	0.000 *X* ^2^ = 17.921
Female	82	42.9	

*Age*			
20–29	106	55.5	0.000 *X* ^2^ = 29.826
30–39	48	25.1	
>39	37	19.4	

*Educational status*			
Diploma	16	8.4	0.000 *X* ^2^ = 77.512
Degree	149	78.0	
Masters	18	9.4	
Others^*∗*^	8	4.2	

*Type of profession*			
Physician	11	5.8	0.000 *X* ^2^ = 60.931
Nurse	87	45.5	
Health officer	9	4.7	
Medical laboratory technician	25	13.1	
Pharmacist	24	12.6	
Midwifery	21	11.0	
Others^*∗∗*^	14	7.3	

*Year of experience*			
<5	134	70.1	0.000 *X* ^2^ = 44.959
5–10	42	22.0	
>10	15	7.9	

*Salary*			
<1500	9	4.7	0.000 *X* ^2^ = 34.214
1500–3500	81	42.4	
3500–5500	67	35.1	
>5500	34	17.8	

^*∗*^Certificate and PhD. ^*∗∗*^Physiotherapist, psychiatrist, anesthetists, radiologist, and optometrist.

## Data Availability

All data generated or analyzed during this study are included in this article and its supplementary information files.

## Supplementary Materials

Supplementary Materials Supplementary materials include the questioner used for data collection with 6 items related to sociodemographic characteristics of participants, 10 items related to telemedicine knowledge, and 22 items related to attitude toward telemedicine.
