# Supplementary material for: Knowledge and Attitude of Health Professionals toward Telemedicine in Resource-Limited Settings: A Cross-Sectional Study in North West Ethiopia
Source: J Healthc Eng. 2018 Nov 18;2018:2389268. doi: 10.1155/2018/2389268 (PMC6276438; doi:10.1155/2018/2389268)
Supplement: Supplementary Materials — include the questioner used for data collection with 6 items related to sociodemographic characteristics of participants, 10 items related to telemedicine knowledge, and 22 items related to attitude toward telemedicine. [file 2389268.f1.pdf]

This questionnaire is to asses Health Professionals knowledge and attitude towards telemedicine. This research is approved by the Research Ethics committee from Debre Markos University. You are selected randomly to participate in this study and your participation is purely based on your willingness. You have the right to choose not to take part in this study and you have the right to stop at any time. All information will be recorded anonymously so please don't write your name or any identification.

Thank you for your time!

Health Professionals knowledge and attitude assessment on telemedicine questionnaire

Table 1. Sociodemographic information of the health professionals

No	question			
1	Sex	male		
		female		
2	Age			
3	Education status	diploma		
		degree		
		masters		
		others		
4	Type of profession	Physician		
		Nurse		
		Health officer		
		Medical laboratory		
		Pharmacist		
		midwifery		
		others		
5	Year of experience	<5		
		5-10		
		>10		
6	salary	<1500		
		1500-3500		
		3500-5500		
		>5500		

Table 2. Health professionals Knowledge toward telemedicine technology

no	Question	Yes	NO			
1	Have you ever heard about telemedicine					
2	If yes, what was your source of information?	training ☐	Public media ☐	internet ☐	colleagues ☐	others ☐
3	Have you ever seen telemedicine system?					

4	I know telemedicine technology		
5	I know telemedicine tools like tele surgery, teleconsultation, teleconferencing and so on		
6	I know the effect of telemedicine on healthcare quality		
7	I know the effect of telemedicine on reducing medical staffs needed		
8	I know about telemedicine infrastructure		
9	I know the benefits of telemedicine on reducing the unnecessary transportation cost		
10	I know the benefits of telemedicine in saving clinicians time		

Table 3. Health professionals' attitude toward telemedicine technology

No	I believe that telemedicine may ….	strongly disagree	disagree	Neutral	agree	strongly agree
	Relative advantage					
12	Reduce medical errors					
13	Facilitate diagnosis and treatment					
14	Increase communication among health care providers					
15	telemedicine can reduce the number of visits to healthcare centers					
16	Enables me accomplish my task more quickly					
17	Improve clinical decisions					
18	Provide more comprehensive healthcare services					
	compatibility					
19	In my opinion, telemedicine is compatible with all aspects of my work					
20	telemedicine is completely compatible with my current situation					
21	I think telemedicine fits well with the way I like to work					
22	Using telemedicine fits well into my work style					
	complexity					
23	I believe using telemedicine requires a lots of mental effort *					

24	Learning to operate telemedicine is hard for me*					
25	I think telemedicine Increase staff work load *					
26	I think telemedicine Create new responsibilities for staff*					
27	In my opinion, telemedicine Threaten information confidentiality and patient privacy*					
	Trial ability					
28	I believe to try telemedicine applications is a great opportunity					
29	I do not have to take very much effort to try out telemedicine					
30	I believe, using telemedicine on a trial basis is enough to see what it could do					
31	I would like to try out telemedicine applications before using it					
	observability					
32	I have seen what other hospital staffs do with telemedicine					
33	telemedicine is very visible in the hospital where I work					
34	In the hospital, I see telemedicine being used for many tasks					

**reverse scored (1=strongly agree ….5=strongly disagree)*
